# Multifaceted Mechanisms of Areca Nuts in Oral Carcinogenesis: the Molecular Pathology from Precancerous Condition to Malignant Transformation

**DOI:** 10.7150/jca.29765

**Published:** 2019-07-08

**Authors:** Yi-Chen Li, Ann-Joy Cheng, Li-Yu Lee, Yu-Chen Huang, Joseph Tung-Chieh Chang

**Affiliations:** 1 Division of Cardiology, Department of Internal Medicine, Kaohsiung Chang Gung Memorial Hospital and Chang Gung University College of Medicine, Kaohsiung 833, Taiwan.; 2 Department of Medical Biotechnology and Laboratory Science, College of Medicine, Chang Gung University, Taoyuan 333, Taiwan; 3 Department of Radiation Oncology, Chang Gung Memorial Hospital-Linkou, Taoyuan 333, Taiwan; 4 Department of Pathology, Chang Gung Memorial Hospital-Linkou, Taoyuan 333, Taiwan; 5 Department of Oral Maxillofacial Surgery, Chang Gung Memorial Hospital-Linkou, Taoyuan 333, Taiwan; 6 Department of Radiation Oncology, Xiamen Chang Gung Memorial Hospital, Xiamen, Fujian, China

**Keywords:** areca nut, oral cancer, oral submucosal fibrosis, reactive oxygen species, tissue hypoxia, cell invasion, epithelial-mesenchymal transition, cancer stemness

## Abstract

Oral cancer is one of the most frequent malignant diseases worldwide, and areca nut is a primary carcinogen causing this cancer in Southeast Asia. It has been widely reported that areca nut induced several cytotoxic effects in oral cells, including ROS generation, inflammation, tissue hypoxia, DNA damage, and cell invasion. Recently, through chronic exposure model, more extensive pathological effects due to areca nut have been found. These include the induction of autophagy, promotion of epithelial- mesenchymal transition, and facilitation of cancer stemness conversion. Clinical findings support these adverse effects. Oral submucosal fibrosis, a premalignant condition, is prevalent in the area with habitual chewing of areca nuts. Consistently, oral cancer patients with habitual chewing areca nut exhibit more aggressive phenotypes, including resistance to chemo-radiotherapy. In this review, we comprehensively discuss and concisely summarize the up-to-date molecular and cellular mechanisms by which areca nuts contribute to malignant transformation. This review may provide critical information regarding clinical applications in risk assessment, disease prevention, diagnosis, and personalized therapeutics for areca nut-induced oral malignancy.

## 1. Introduction

Oral cancer, including the common squamous cell carcinomas of the oral cavity and oropharynx, is the sixth most frequent cancer worldwide 1, 2. The disease is more prevalent among males than females 1, 2. Epidemiologic studies have shown wide variations in its incidence in different geographical areas. Oral cancer is highly prevalent in Southeast Asia, comprising 35-40% of all malignancies in India, compared to approximately 9% in Taiwan and 2-4% in western countries 1-4. The tumor sites of this disease differ in different geographical regions. Cancers of the tongue and buccal mucosa constitute the majority of oral cancers in India and Southeast Asia 1-4. In contrast, the western regions show that cancers of the mouth floor are the most frequent, with cancer of the gums or tongue being rare 1-4. Apparently, oral cancer also shows various clinicopathological features in different global regions.

The association of carcinogen exposure with oral cancer has been reported 3-5. Carcinogens include habitual alcohol consumption, areca nut chewing, and cigarette smoking. Cigarette smoking and alcohol consumption are common habits in oral cancer patients in western countries. Cigarette smoking may render a significant carcinogenic effect on the upper aerodigestive tract, including oral areas 3-5. Areca nut chewing is a common habit among oral cancer patients in Southeast Asia, indicating a close link of this habit with the specific disease. In Taiwan, for example, approximately 85% of all oral cancer patients are associated with this habit 3, 4. It has been shown that areca nut chewers have a much greater risk of developing oral cancer than nonchewers 3-6. Furthermore, the 5-year survival rate of oral cancer patients who chew areca nuts is much lower than that of those who do not chew these nuts 7, 8. Therefore, the distinct clinicopathological characteristics of oral cancer in global regions may result from different environmental carcinogenic exposures in addition to genetic factors. In this paper, we comprehensively review and concisely summarize recent reports of the molecular and cellular effects of areca nuts that lead to the development of oral cancer.

## 2. Molecular pathology of areca nut

### 2.1 Areca nuts are a primary carcinogen in Southeast Asia, with several active components

Areca nuts have been one of the most commonly used psychoactive substances for a century, especially in Southeast Asia. For better chewing flavor in using, areca nuts are usually covered with piper betel leaves or inflorescence to form betel quids 3, 4. Areca nut extract is composed of saccharides (26-47%), polyphenols (11-26%), fats (1.3-17%), various alkaloids (0.15-0.67%), and some crude fiber and rare tannins 4-6. Although alkaloids constitute only a few percent of all components, they are the most active ingredients associated with pathological development. Arecoline is the most abundant alkaloid, and it can be converted to arecaidine by salivary enzymes 4,6. These alkaloids are converted to nitroso-derivatives, the primary inducers of oral mucosal lesions 4,6.

In the past few decades, accumulated studies have demonstrated that areca nuts can induce premalignant and malignant transformation of oral tissues. In animal model studies, areca nut extract (or the ingredient cocktail) can be an effective tumor initiator or promoter and can induce premalignant oral lesions, including submucosal fibrosis 9, 10 and squamous hyperplasia 11-13, or result in malignant transformation 13-16. For example, arecaidine displays a synergistic effect in the 7,12-dimethyl-benz(a)anthracene (DMBA)- induced tumor formation in the cheek pouch of hamster 15. Similarly, combinational exposure of arecoline and 4-nitroquinoline-1-oxide (4-NQO) induces oral cancerous lesions in C57BL/6JNarl mice 11, 16. Since the carcinogenic effect of areca nuts has become more visible, in 1992, the International Agency for Research on Cancer (IARC) announced that areca nut chewing combined with cigarette smoking is a human carcinogen. In 2004, IARC announced that the areca nut itself is a human carcinogen 17.

### 2.2 Oral submucosal fibrosis is a common premalignant disorder induced by areca nut chewing

The carcinogenesis of oral cancer is a multiple-step progressive process 6. It is started from normal epithelial cells, gradually evolving to premalignant lesions. After malignant transformation, these cells eventually become aggressive types of cancers. Clinically, the premalignant oral disorders can be classified by distinct pathological features. These include hyperkeratosis, dysplasia, leukoplakia, erythroplakia, and fibrosis. Among these, leukoplakia is the most common disorder, while erythroplakia although rare, is more serious 18. Both leukoplakia and erythroplakia are considered as premalignant lesions 18. Oral submucosal fibrosis is a chronic progressive process, presenting an inflammatory fibrosis in oral mucosa stroma, being considered as premalignant condition 19-21. This disorder is prevalent in India and Southeast Asia, a common premalignant condition caused by prolonged areca nut chewing 18-22. Approximately 18% of the premalignant oral lesions will develop into squamous cell carcinoma 3. Transformation of oral submucosal fibrosis may be variable, begin estimated between 2% to 8% and up to 13% 20-22.

The pathological effect of areca nut contributing to oral submucosal fibrosis is supported by several lines of studies. In a mouse model, the subcutaneous injection of areca nut extract-induced skin lesions and fibrosis 11. This is accompanied with the expression of fibrotic marker proteins including alpha-smooth muscle actin (α-SMA) and connective tissue growth factor. In a cellular study, treatment of oral keratinocytes with arecoline upregulated the expression of αvβ6 integrin and α-SMA to promote the formation of submucosal fibrosis 23, 24. Treatment of fibroblast cells with areca nut extract also induced cell contraction mediated by several signaling pathways, including the JNK/ATF2/Jun axis, Ca2+/calmodulin axis, and Rho protein activation, leading to actin filament polymerization 23, 25.

The accumulation of collagen is a notable pathological alteration in the patients with oral submucosal fibrosis 26. In fibroblast cells, arecoline increased collagen expression by 1.5-fold 27, and this might result from elevating TIMP-1 and inhibiting gelatinase A activity 28. Other areca nut components, such as flavonoids, catechins and tannins, facilitate the crosslinking of collagen fibers, resulting a decreased susceptibility to collagenase 29. Increases in transforming growth factor β1 (TGF-β1) during areca nut-induced oral submucosal fibrosis has also been observed in many studies 10, 23, 30. In the condition of submucous fibrosis, TGF-β plays a critical role in regulating the degradation of the extracellular matrix, including collagen 31. Consistently, it has been shown that arecoline augments collagen levels through the TGF-β pathway in keratinocytes 32. In summary, the areca nut promotes oral fibrosis by increasing collagen production, which may occur by various mechanisms, including reduction of degradation by proteinase and activation of the TGF-β regulatory pathway.

The malignant transformation from oral submucosal fibrosis to squamous carcinoma involves multifactorial mechanisms. These include inflammation, genotoxicity, cytotoxicity, autophagy, tissue hypoxia, and epithelial-mesenchymal transition (EMT), as discussed respectively in the following sections.

### 2.3 Chewing areca nuts increases ROS levels and induces inflammation

Reactive oxygen species (ROS) are chemically reactive chemical species containing oxygen that can be generated during mitochondrial oxidative metabolism.

Under environmental stress, cellular ROS levels can become dramatically elevated and directly cause significant damage to cell structures at DNA, protein, or lipid levels 33. Many studies have demonstrated that areca nuts can stimulate cellular ROS levels 34. The mechanisms include the enhancement of ROS generation by mitochondrial metabolizing enzymes, such as cytochrome P450s (CYPs) 35, by the NADPH oxidase enzymes NOX-1 and NOX-4 36, and the inhibition of antioxidant systems by the suppression of superoxide dismutase activities 37, 38.

The induction of ROS by areca nut extract has been further shown to act as a molecular signal to elicit redox-related inflammation or signaling pathways in many types of cells. For example, in endothelial cells, arecoline stimulates ROS production to suppress the expression of the cytoprotective enzyme hemeoxygenase-1 39. In lymphocytic cells, areca nut extract elicits oxidative stress and inflammatory responses and upregulates several cytokines, including NF-kB, Cox-2, PGE2, TGM2, and IL-1 40. Similarly, in fibroblasts and keratinocytes, areca nut extract or arecoline triggers ROS generation and induces tumor promoting mediators or oncogenic signaling pathways, including IL-6, TGF-b, EGFR, ERK and Ras 36, 41-43. The different cytokines or signaling pathways in response to areca nut treatment may be cell type-specific. Clinically, the inflammation associated cells are increased in the surrounding tissues of oral submucosal fibrosis and oral cancers in the patients from areca nut chewing prevalent area 44. In summary, areca nut extract may increase ROS levels, which facilitate cellular inflammation and tumor progression via multiple molecular regulators.

### 2.4 Areca nuts may elicit genotoxicity, growth arrest, and apoptosis

Many studies have demonstrated that areca nut extract exerts multiple cellular effects. Genotoxicity can be caused by areca nut exposure. The alkaloids of the areca nut are the major contributing factors to genotoxicity 6. In oral keratinocytes or epithelial cells, areca nut extract or arecoline can induce genetic damage, including hyperdiploid chromosomal changes 45-47. These DNA damage effects are associated with the inhibition of DNA repair mechanism, such as impairing p53 function 46, 47. In a mouse model study, arecadine increased the frequency of sister chromatid exchanges during mitosis 48. In a transgenic mouse study, arecoline increased the frequency of mutations at DNA G:C sites in oral tissue cells 49. These genotoxic results are consistent with clinical findings in betel quid chewers. In the oral mucosal cells, the levels of chromosome damage, such as cytokinesis-block micronucleus, chromatid breaks or DNA strand exchanges, are positively correlated with habitual areca nut chewing in oral cancer patients 50-52. Note that these cytogenetic alterations are less frequently observed in oral cancer patients who chew tobacco 51, 52.

Areca nuts may cause cytotoxicity in various types of human tissues and result in growth arrest, cellular senescence or apoptosis. In oral endothelial cells, arecoline induces G2/M cell cycle arrest and increases the sub-G0/G1 population, suggesting causal links among endothelial damage, vascularity reduction, and the pathogenesis of oral submucosal fibrosis 53, 54. Similarly, in oral keratinocytes, epithelial cells, or neutrophils, areca nut ingredients or arecoline may contribute to G1/S cell cycle arrest, cellular senescence or apoptosis 37, 42, 55-59. These effects may be caused by the activation of various signaling pathways, including Chk1/Chk2, MEK/ERK or AKT associated pathways 42, 59. The different cytotoxic effects and molecular pathways in response to areca nut stimulation may be dependent on specific cell types or the differential microenvironmental factors. In all, growth inhibition or cell suicide may be the optimal cellular defense mechanism to avoid further catastrophe of malignant transformation.

### 2.5 Areca nuts may induce autophagy and inhibit tumor suppressors

Although areca nut ingredients may lead to growth arrest and cell apoptosis, prolonged treatment with areca nut extract or arecoline may further facilitate malignant transformation. This is presumably via the induction of cellular autophagy or the inhibition of tumor suppressors. Autophagy is a process by which cells degrade unnecessary organelles and recycle intracellular proteins to ensure survival in adverse environments. Although autophagy may play dual roles in carcinogenesis, in most contexts, it promotes tumorigenesis 60. The premalignant submucous fibrotic tissue or cancer cells may upregulate autophagy to survive microenvironmental stress and become more aggressive 4, 61. Recent reports have shown that areca nut extract may induce autophagy via several pathways. For example, areca nut ingredients may be engulfed by oral cancer cells via clathrin-mediated endocytosis to initiate an autophagy program 62. The other autophagy associated mechanisms, such as the LC3-II transition, Beclin-1 or Atg5 accumulation, and autophagosome formation, have also been demonstrated in oral cancer cells treated with areca nut extract 62-64. The contribution of areca nut extract to autophagy may be explained by ROS generation or hypoxic condition in cancer cells, following the stimulation by various signaling pathways of PI3/AKT, MEK/ERK, AMPK/mTOR, or HIF-1α 63, 65-66. Clinically, higher LC3 expression and poorer prognoses are found in advanced oral cancer patients who habitually chew areca nuts 66.

In addition to autophagy induction, areca nuts may inhibit tumor suppressor molecules to promote malignant transformation. It has been demonstrated that areca nut extract or arecoline may inhibit the expression of cell cycle checkpoint suppressors, including p53, p21, p27, and Ches1, which may enable cell cycle progression with error-prone DNA replication 46, 67, 68. Clinical findings also support this concept. The reduced expression of the Ches1 suppressor has been found in oral cancer patients with the areca nut chewing habit 67. In summary, the areca nut may contribute to cellular transformation by activating cellular stress response mechanisms, such as ROS generation, autophagy formation, and tumor suppressor inhibition.

### 2.6 Area nuts may induce tissue hypoxia to promote malignant transformation

Hypoxia is a low oxygen stress condition in tissue microenvironment, giving rise to altered cellular metabolism and triggers various pathophysiological responses 69. This condition may be associated with the cellular oxidative stress 65, 69, and induce anaerobic respiratory pathway via up-regulations of hypoxia inducing factor (HIF), glucose transporter (GLUT), or lactate dehydrogenase 70-72. Currently, hypoxia has been shown as an important underlying factor to promote tumorigenesis and cancer progression. It may incite several cellular mechanisms including autophagy, angiogenesis, epithelial to mesenchymal transition, cancer stemness, and lead to therapeutic resistance 73-74. For examples, in oral submucous fibrosis, the tissue hypoxia resulted from vascular construction may further facilitate malignant transformation 20-22, 71. Tissue hypoxia may further trigger EMT process via up-regulation of several transcriptional factors, such as Snail and Twist1, to promote tumor progression 75-76. In cancer cells, the induction of HIF-1α molecule in the adaptation of hypoxic condition may elite angiogenic pathway via up-regulation of vascular endothelial growth factor (VEGF) 77. Hypoxic condition in tumor microenvironment may also lead to therapeutic resistance by activation of stemness associated pathways, including Oct3/4, Sox2, and AKT/Notch1 molecular signals 78, 79.

The hypoxia inducing factor-1 (HIF-1) is a predominant regulatory molecule induced by hypoxia tissue and emerges to malignant function 72, 73, 77. Recent reports have shown that areca nut causative to oral malignancy may associate with the hypoxic condition through the induction of HIF-1. In either oral fibroblast or oral cancer cells, arecoline may increase HIF-1α gene expression with a dose-dependent manner 80, 81. In oral cancer cells, treatment of areca nut extract may induce ROS generation and up-regulate HIF-1α, which may further lead to autophagy to benefit cell survival 65. The prolong treatment of areca nut extract in oral cancer cells results to a stronger tolerance in hypoxic condition via acquisition of autophagy and leads to chemoresistance 82. Consistently, under a stress condition, areca nut extract induced VEGF expression in oral cancer cells, suggesting the mechanism of areca nut contributes to angiogenesis and cancer metastasis 83. Clinically, higher levels of HIF-1α and PAI-1 have been found in the tissues of oral submucosa fibrosis or oral cancer cells compared to the normal mucosa 81, 84. In summary, areca nut may incite tissue hypoxia to promote malignant transformation via multiple mechanisms.

### 2.7 Areca nuts may promote cell motility and epithelial-mesenchymal transition

Cell motility is an important characteristic of the malignancy response for cancer invasion and metastasis. In cells, the matrix metalloproteinases (MMPs) constitute a family of proteinases that degrade the extracellular matrix to accelerate cellular motility and invasion, whereas the tissue inhibitors of the metalloproteinases (TIMP) counteract this enzymatic activity. The areca nut contributes to oral malignancy by promoting cell motility as well. It has been widely reported that treatment with areca nut extract or arecoline can increase cellular migration, invasion or anchorage-independent growth in oral cancer cells, normal epithelial cells and fibroblast cells 85-91. This response may result from the elicitation of MMP activities, including MMP-1 80, 91, MMP-2 81, 90, MMP-8 85, and MMP-9 59, 88, 89, and the suppression of TIMP functions 59, 92. Multiple molecular signaling pathways, such as PI3K, p38 MAPK, Erk1/2, and NF-kB may be involved in the modulation of MMP and TIMP expression 87, 88, 93 and may through the muscarinic M4 receptor 93. Clinical findings support this cell motility mechanism. High levels of MMP-1 or MMP-9 are found in the cancer tissues or saliva specimen of oral cancer patients who chewed betel nuts 85, 86, 93. Apparently, the areca nut promotes cell motility through MMP activation, although different MMP proteins may respond differentially in different individuals.

The epithelial-mesenchymal transition (EMT) plays an important role in cell motility conversion leading to cancer aggressiveness 94-97. The EMT confers tumor plasticity by transforming epithelial cells into spindle-like fibroblastic mesenchymal cells via functional loss of cell adhesion and the acquisition of migratory properties 94, 95. This process involves disassembling cell-cell and cell-matrix junctions by downregulating epithelial markers (such as E-cadherin) and upregulating mesenchymal markers (such as N-cadherin) 94, 95. It may be induced by several EMT/stemness associated transcription factors, allowing cells to gain stemness-related properties and create a pro-tumorigenic setting 96, 97. Recent studies show that areca nut extract may induce oral fibrogenesis and carcinogenesis through EMT process. In buccal mucosa fibroblasts, areca nut extract or arecoline induces fibroblast trans-differentiation, which may be mediated by EMT associated transcription factors ZEB1, Twist, and Slug 98-100. This fibrotic activity was found accompanying with collagen gel contraction, increase of marker protein expression (α-SMA), and elevation of migration capability 99, 100. Similarly, in gingival fibroblasts or epithelial cells, areca nut stimulates fibrotic activation, preassembly through induction of EMT process via TGF-β signaling pathways 23, 101, 102. Consistently, in either oral keratinocytes or cancer cells, areca nut facilitates EMT process, as shown by the increases of mesenchymal markers (N-cadherin, vimentin) and decreases of epithelial markers (E-cadherin, involucrin), via activating the PI3/AKT pathway 103, 104. In oral epithelial or cancer cells, chronic or long-term treatment of areca nut extract facilitates mesenchymal trans-differentiation, along with the induction of multiple EMT associated transcription factors, including ZEB1, Snail, Slug, Twist, FOXC2, and Grp78 86, 105, 106. Furthermore, a keratin family member, Krt-17, was found to be upregulated by areca nut extract to facilitate cell motility and malignant transformation via EMT conversion in a mouse model study 11. Clinically, the expression of EMT associated factor Slug has been found up-regulated in oral fibroblastic tissues and associated with various myofibroblast markers, such as α-SMA 99, 100. The loss of E-cadherin expression and augmentation of Krt-17 or EMT-associated transcription factors have also been shown to be significantly associated with oral cancer in patients who habitually chewed betel quid 11, 86, 107.

### 2.8 Areca nuts may facilitate chemo-radioresistance and cancer stemness conversion

Chemotherapy and radiotherapy are integral parts of the treatment for oral cancer. However, local recurrence after radio-chemotherapy is a major cause of therapeutic failure. Although areca nuts may elicit genotoxicity leading to cell death, recent studies showed that chronic areca nut exposure may eventually result in chemo- and radio-resistance. In oral cancer cells or normal keratinocytes, long-term exposure to arecoline or areca nut extract resulted in higher tolerance to cisplatin or fluorouracil 82, 86, 105. This areca nut-induced drug resistance may be attributed to overexpression of the ABCG2 protein, a well-known drug efflux pump in cancer cells 86. Consistent with these reports, oral cancer cells chronically exposed to areca nut extract exhibited greater resistance to irradiation 86. This survival advantage is accompanied by the reduction of ROS production by the elevation of the scavenger enzymes GCLC and GCLM 86. Similarly, in keratinocytes, treatment with sublethal doses of arecoline upregulated the expression of multiple antioxidant enzymes, including G6PD, GCLC and glutathione reductase 108. Clinically, the habitual use of areca nuts is an independent prognostic factor of poor survival of oral cancer patients receiving induction chemotherapy with docetaxel, cisplatin, or fluorouracil 8, 109. The ERCC1 molecule, a critical DNA repair gene associated with chemoresistance, is up-regulated in oral cancer patients in the areca nut prevalent area 109. Similarly, oral cancer patients who chew areca nuts habitually exhibit higher incidences of local recurrence 7. Thus, chronic exposure to areca nuts facilitates chemo-radioresistance, which may due to the activation of cellular defense mechanisms to minimize the toxic damage in malignant transformed cells.

A cancer stem cell (CSC) model has been recently proposed to explain tumor heterogeneity. These cells, although comprise a small fraction within a tumor, possess a strong malignant potential, with self-renewal ability, stress tolerance, and high mobility, which results in aggressive cancer phenotypes and resistance to chemo-radiotherapy 110-112. These types of stem-like cells are often characterized by specific surface proteins, such as CD44, CD133 and ALDH1, in oral cancer tissues 86, 113, 114. Recent reports provide new insights that areca nuts may play a role in cancer stemness conversion. In oral cancer cells or normal keratinocytes, chronic areca nut exposure or long-term arecoline treatment facilitates the cancer stemness conversion. These were demonstrated by the enhanced spheroid cell formation and enriched subpopulations of CD24-/CD44+, CD133+, and ALDH1+ cells 86, 105. Furthermore, these chronic areca nut exposures exerted a pluripotent effect to upregulate several stemness mediators, including Grp78, Slug, Snail, Oct4, Nanog, and Sox2 86, 105. This phenomenon is confirmed in clinical investigations. Oral cancer patients who habitually chewed areca nuts exhibited high levels of stemness regulators, such as Grp78 and snail, which was correlated with worse prognoses 86, 115, 116. Consistently, oral cancer patients who habitually chewed areca nuts possessed cancers with more aggressive attributes, including higher incidences of second primary tumors, microsatellite residual tumors, and poor survival; all of which are characteristic of cancers with stemness properties 7, 117. Thus, the areca nut contributes to malignancy by facilitating the conversion of CSCs via multiple stemness regulatory mechanisms.

## 3. Conclusion

**Figure 1:**
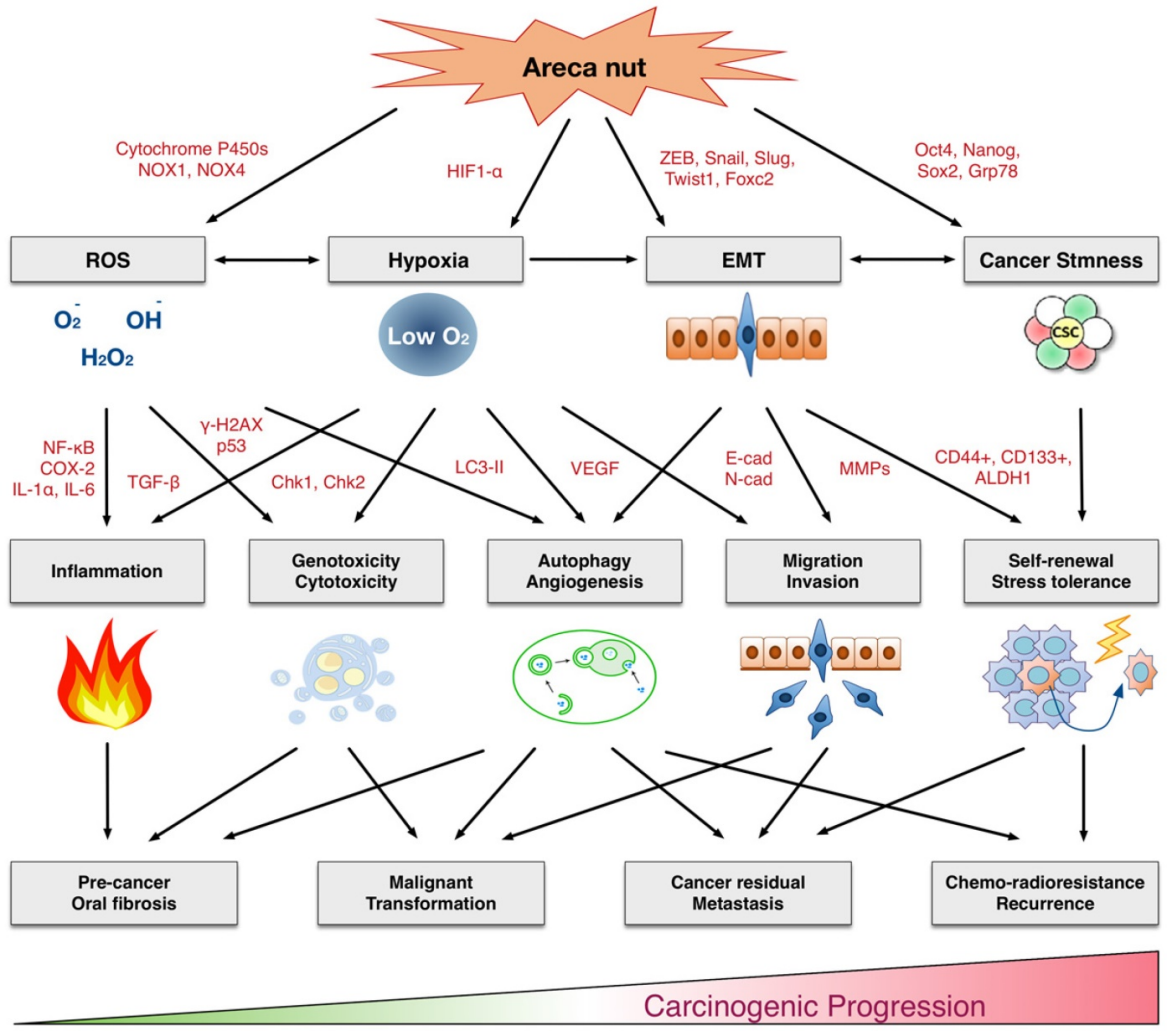
Multifaceted mechanisms of areca nuts in oral carcinogenesis: the molecular pathology from precancerous lesions to malignant transformation. ROS: Reactive oxygen species. EMT: Epithelial-mesenchymal transition.

In this review, we comprehensively discuss the underlying molecular and cellar mechanisms by which areca nuts contribute to malignant transformation. As summarized in Figure 1, the areca nut induces multiple cytotoxic effects, including inflammation, tissue hypoxia, DNA damage, autophagy, invasion, and chemo-radioresistance. These cellular effects are accompanied by numerous molecular alterations involving the production of reactive oxygen species, activation of various signaling pathways, promotion of epithelial-mesenchymal transition, and facilitation of cancer stemness conversion. Clinical findings support these adverse effects. Oral submucosal fibrosis is prevalent in the area with habitual chewing of areca nuts. The oral cancer patients who habitually chewed areca nuts exhibited more aggressive cancer phenotypes, with higher rates of cancer metastasis, recurrence, and poor patient survival. Thus, areca nuts contribute to oral carcinogenesis via multifaceted mechanisms. This review may provide critical information for the risk assessment, disease prevention, diagnosis, and personalized or molecular therapeutics for clinical applications in areca nut-induced oral malignancy.
